# Ocular Mucosal CD11b+ and CD103+ Mouse Dendritic Cells under Normal Conditions and in Allergic Immune Responses

**DOI:** 10.1371/journal.pone.0064193

**Published:** 2013-05-14

**Authors:** Payal Khandelwal, Tomas Blanco-Mezquita, Parisa Emami, Hyun Soo Lee, Nancy J. Reyes, Rose Mathew, Randy Huang, Daniel R. Saban

**Affiliations:** 1 Schepens Eye Research Institute, Massachusetts Eye and Ear Infirmary, Department of Ophthalmology, Harvard Medical School, Boston, Massachusetts, United States of America; 2 Department of Ophthalmology, Duke Eye Center, Duke University School of Medicine, Durham, North Carolina, United States of America; 3 Department of Immunology, Duke University School of Medicine, Durham, North Carolina, United States of America; Oklahoma Medical Research Foundation, United States of America

## Abstract

Steady state dendritic cells (DC) found in non-lymphoid tissue sites under normal physiologic conditions play a pivotal role in triggering T cell responses upon immune provocation. CD11b+ and CD103+ DC have received considerable attention in this regard. However, still unknown is whether such CD11b+ and CD103+ DC even exist in the ocular mucosa, and if so, what functions they have in shaping immune responses. We herein identified in the ocular mucosa of normal wild-type (WT) and Flt3-/- mice the presence of a CD11b+ DC (i.e., CD11c+ MHCII+ CD11b+ CD103- F4/80+ Sirp-a+). CD103+ DC (i.e. CD11c+ MHCII+ CD11b low CD103+ CD8a+ DEC205+ Langerin+) were also present in WT, but not in Flt3-/- mice. These CD103+ DC expressed high levels of Id2 and Flt3 mRNA; whereas CD11b+ DC expressed high Irf4, Csfr, and Cx3cr1 mRNA. Additionally, the functions of these DC differed in response to allergic immune provocation. This was assessed utilizing a previously validated model, which includes transferring specific populations of exogenous DC into the ocular mucosa of ovalbumin (OVA)/alum-primed mice. Interestingly, in such mice, topical OVA instillation following engraftment of exogenous CD11b+ DC led to dominant allergic T cell responses and clinical signs of ocular allergy relative to those engrafted with CD103+ DC. Thus, although CD11b+ and CD103+ DC are both present in the normal ocular mucosa, the CD11b+ DC subset plays a dominant role in a mouse model of ocular allergy.

## Introduction

As the most potent stimulators of T lymphocytes, dendritic cells (DC) are widely appreciated as a very unique subpopulation of antigen presenting cells and key in the generation, as well as the regulation, of adaptive immune responses. Much of their proficiency in T cell priming is attributed to high-level expression of MHC class II and costimulatory molecules such as CD80 and CD86. In addition, steady state DC, which take up residence in uninflamed non-lymphoid tissues such as the mucosa, are also highly efficient in mobilizing to lymphoid organs for Ag presentation to T cells.

Now fully appreciated is the understanding that DC comprise multiple populations with differing ontogenies [1]. Two recently described subsets receiving considerable attention include, CD11b+ DC (i.e., CD11c+ MHCII+ CD11b+ CD103 low) and CD103+ DC (i.e., CD11c+ MHCII+ CD11b low CD103+) [1]–[5]. These cells have been described in mucosal tissues lining the lung and gastrointestinal tracts, in addition to tissues of other organs, e.g. liver and pancreas [1]–[5]. CD11b+ DC can be identified via their F4/80+ Sirp-a+, while CD103+ DC are CD8a+ DEC205+ langerin+ [2]–[4]. Transcriptional programs are also disparate, as CD11b+ DC express Irf4, Csfr, and Cx3cr1 [2]–[4], whereas CD103+ DC express Irf8, Id2, and Flt3 [2]–[4]. It is widely agreed that CD103+ DC arise from pre DC precursors in an Flt-3 dependent manner, whereas CD11b+ DC may be a heterogeneous population derived from multiple precursors, including pre DC and monocytes [2], [3]. In addition, a third population has also been described in the gut lamina propria, which is CD103+ CD11b+ double positive [1].

Considerable attention has also been focused on attempting to elucidate the precise functional roles of CD103+ and CD11b+ DC; however, many questions still remain. Indeed, numerous reports have converged on the importance of viral Ag cross presentation by CD103+ DC [5]–[8]. In addition, their robust IL-12 production [9] has led to the tenet that CD103+ DC are preferential for the generation of Th1 responses. However, this has been challenged by reports demonstrating an importance for CD103+ DC in tolerance [10] and homeostasis [11]. Likewise, a recent report by Nakano et al demonstrated that pulmonary CD103+ DC are necessary to prime Th2 responses to inhaled allergens [12]—a finding which also challenges the notion that CD11b+ DC are preferential to stimulation of Th2 responses [13]–[15]. Thus, future work is required in order to further elucidate the nature and function of steady state CD103+ versus CD11b+ DC in adaptive immunity.

Many of the mucosal tissue sites (e.g., airway and gastrointestinal tracts) have already been probed for identification and function of CD11b+ and CD103+ DC, with an exception for the ocular mucosa (i.e., bulbar, fornix and palpebral conjunctiva) [1]–[5]. Investigating this would also be important in the ocular mucosa, which can indeed suffer from immunologic pathologies including those of autoimmune (e.g., conjunctival cicatrization), infectious (e.g., herpetic conjunctivitis) and/or allergic (e.g., atopic keratoconjunctivitis) origins. Steady state Langerhans cells and langerin+ DC have been identified in the normal mouse cornea [16], but have not been reported in the conjunctiva (conj). However, Ohbayashi et al have identified CD11c+ CD11b+ cells in the mouse ocular mucosa via immunohistochemistry [17], [18]. Whether the lineage and phenotype of such CD11b+ DC are akin to steady state CD11b+ DC previously described [2]–[5], and if CD103+ DC also exist there, is completely unknown.

We therefore investigated this in the current study, and were able to determine that CD11b+ and CD103+ DC exist in the ocular mucosa of mice. Interestingly, use of a previously described model of ocular allergy, whereby exogenous DC (e.g., CD103+ versus CD11b+ DC) are engrafted into the ocular mucosa in topically challenged mice [19], revealed that CD11b+ DC were dominant in triggering allergic T cell responses and clinical signs of ocular allergy. Thus, we demonstrate herein that CD103+ and CD11b+ DC reside in the ocular mucosa; however, they appear to play different roles in mediating adaptive immune responses relevant in allergy.

## Results

### Characterization of steady state DC subsets of the ocular mucosa in wild type and Flt-3 KO mice

**Figure 1:**
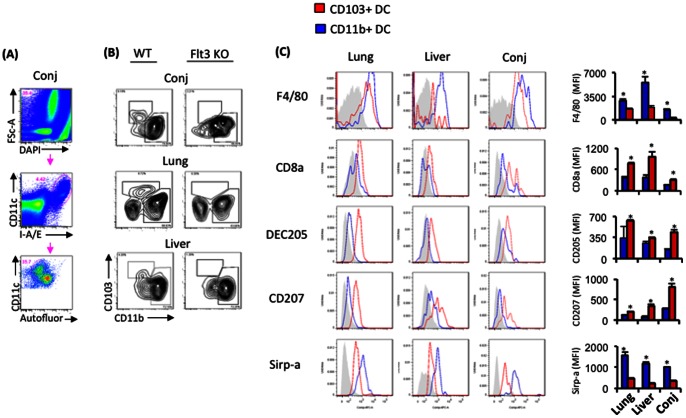
DC subsets in the ocular mucosa are consistent phenotypically with CD103+ and CD11b+ DC. (**A**) Gating scheme for analyses of conj DC subsets. Single cell preparations of harvested conj were analyzed for expression of the indicated markers. Events were gated on for viable (DAPI-), singlet (data not shown), CD11c+, I-A/E+, and autofluorescent- (empty FITC channel) cells, and then examined for CD11b+ and CD103+ DC subsets. (**B**) The presence of steady state CD103+, but not CD11b+, DC in the ocular mucosa is Flt-3 dependent. The above gating scheme was used to analyze the presence of CD11b+ vs. CD103+ DC from lung, liver and conj of WT and Flt-3 KO mice. (**A-B**) This experiment was derived from pooled lung (n = 3 mice), liver (n = 3 mice), and conj (n = 10). (**C**) DC subsets of the ocular mucosa share a uniform phenotype with steady state CD103+ and CD11b+ DC. The aforementioned gating scheme was used to analyze CD11b+ vs. CD103+ DC and the mean fluorescence intensities (MFI) of indicated markers were plotted. Statistical significance (*p<0.05) was calculated by comparing MFI of CD11b+ vs. CD103+ DC from pooled lung (n = 3 mice), liver (n = 3 mice), and conj (n = 10). (**A–C**) Data shown are representative of 3 independent experiments.

Steady state CD11b+ and CD103+ DC were previously identified in numerous tissues of the mouse, including the liver and lung [1]–[5]. CD103+ DC arise from pre DC progenitors in a Flt-3 dependent manner; whereas, CD11b+ DC may partially arise from this pathway, as well as from monocyte precursors in a Flt-3 independent manner [1]–[5]. In the current study, we used flow cytometry to examine the ocular mucosa, i.e. conj, for the presence of distinct CD103+ and CD11b+ DC subsets, and compared these with known CD103+ and CD11b+ DC previously described in the liver and lung [1]–[5]. We also queried Flt-3 KO mice to this end, which have been previously shown to be absent of CD103+ DC in numerous organs such as the liver and lung [1]–[5]. In our study, we identified, similar to the liver and lung, the presence of a distinct population of CD103+ DC (CD11c+ I-A/I-E+ autofluorescent- CD11b low/neg CD103+) and another population of CD11b+ DC (CD11c+ I-A/I-E+ autofluorescent- CD11b+ CD103 low/neg) in the conj (**Fig. 1a, b**). The frequency of CD11b+ DC was greater than that of CD103+ DC for all tissues evaluated (**Fig. 1b**). Furthermore, as seen in the liver and lung, conj of Flt-3 KO mice were absent of CD103+, but not CD11b+ DC (**Fig. 1b**).

### Ocular mucosal DC subsets express phenotypes and transcriptional programs that are consistent with steady state CD11b+ and CD103+ DC

Conclusive identification of CD103+ and CD11b+ DC in the ocular mucosa required further investigation, which we in part accomplished by querying phenotypic expressions unique to these DC subsets [1]–[5]. Based on their monocyte lineage, CD11b+ DC express F4/80 and Sirp-a, while CD103+ DC are associated with the expression of CD8a, DEC205, and langerin [1]–[5]. We pursued such identifications via FACS analyses of conj DC subsets, and compared them directly to previously described CD103+ and CD11b+ DC in the liver and lung [1]–[5]. Tissues were harvested from WT mice and digested into single cells. The aforementioned gating scheme was used to gate CD103+ versus CD11b+ DC, and F4/80, CD8a, DEC205, Langerin, and Sirp-a mean fluorescence intensities (MFI) were analyzed (**Fig. 1c**). In assessing these markers we observed that CD103+ DC of the conj expressed significantly higher levels of CD8a, DEC205, and langerin (**Fig. 1c**); while CD11b+ DC expressed significantly higher levels of F4/80+ and Sirp-a. This trend was similarly observed in the liver and lung (**Fig. 1c**).

**Figure 2:**
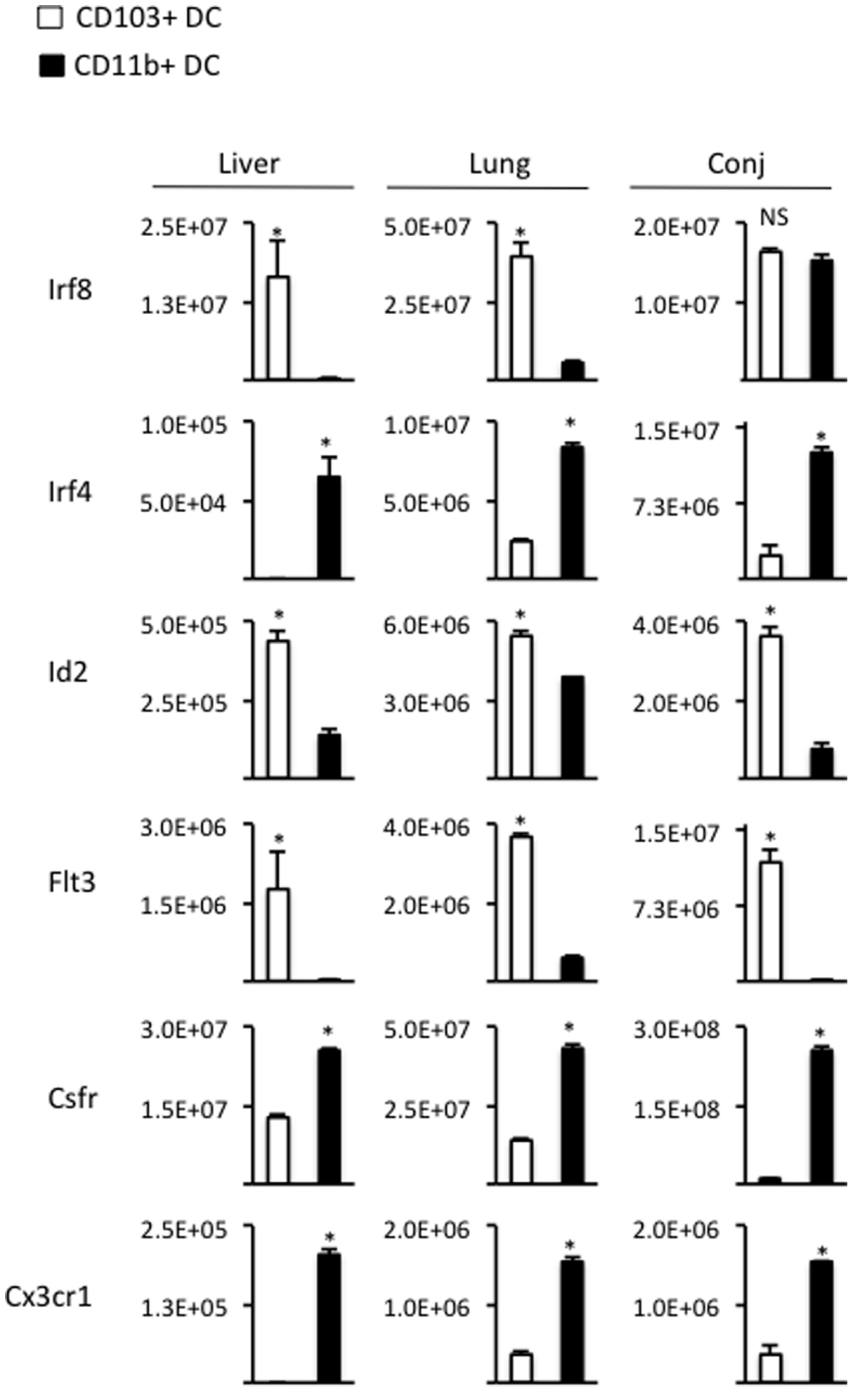
Transcriptional programs expressed by ocular mucosa DC subsets are consistent with steady state CD103+ and CD11b+ DC. Lung, liver and conj from WT mice were prepared into single cell suspensions. Aforementioned gating scheme for FACS sorting was used to isolate CD11b+ vs. CD103+ DC. Indicated genes were assayed for by qRT-PCR assayed in the sorted DC subsets. Statistical significance (*p<0.05) via student's t test was derived from assessing pooled lung (n = 3 mice), liver (n = 3 mice), and conj (n = 25). Data shown are representative of 2 independent experiments.

To further verify our identification of CD103+ and CD11b+ DC in the conj, we also examined unique transcriptional programs expressed by these subsets [1]–[5]. CD11b+ DC express Irf4, Csfr, and Cx3cr1 [1]–[5]; whereas CD103+ DC are associated with Irf8, Id2, and Flt3 [1]–[5]. Thus, in the current study, CD11b+ versus CD103+ DC were FACS sorted from conj, liver, and lung of WT mice using the aforementioned gating scheme. Subsequent analyses relied on qRT-PCR to examine the expressions of Irf8, Irf4, Id2, Flt3, Csfr, and Cx3cr1 in the sorted populations (**Fig. 2**). In doing so, we observed significantly higher levels of Irf4, Csfr, and Cx3cr1 expression by CD11b+ DC in the conj, liver and lung; whereas CD103+ DC expressed significantly higher levels of Id2 and Flt3 (**Fig. 2**). CD103+ DC also showed significantly higher expression of Irf8 in the liver and lung, but not in the conj (**Fig. 2**).

### Examination of CD11b+ versus CD103+ DC in triggering allergic immune responses in a model of ocular allergy

With the conclusive identification of CD103+ and CD11b+ DC in normal conj, we next moved on to assess the role of these respective DC subsets in a model of ocular allergy. We utilized our previously described model to accomplish this [19], which involves adoptive transfer of T cells from OVA/alum-primed mice and topical OVA challenge in hosts which have received exogenously derived DC by their engraftment into the conj. Such site-specific engraftment of DC leads to a robust augmentation of allergic immune responses and provides direct functional information of engrafted DC [19].

**Figure 3:**
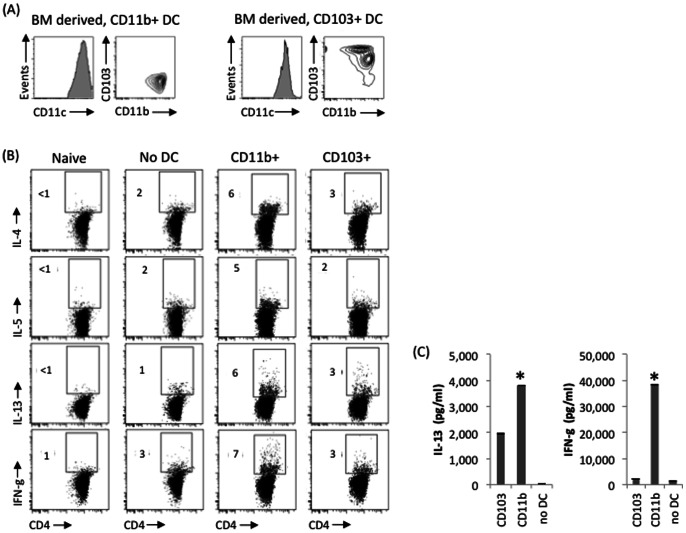
Secondary allergic T cell responses are augmented as a result of conjunctival engraftment of purified BM-derived CD11b+ DC. (**A**) MACS sorted BM derived CD103+ and CD11b+ DC. (**B**) Naïve host mice were adoptively transferred with purified T cells from OVA/alum primed mice. Hosts were subsequently engrafted with purified BM-derived CD103+ or CD11b+ DC (1.0×10ˆ5 cells), or sham engrafted with HBSS, prior to OVA ocular instillations. T cells from eye draining LN were harvested and stimulated in vitro with OVA for subsequent intracellular flow cytometry analyses of the indicated cytokines. This experiment is derived from pooled LN collected from an n = 4 mice per group. Data are representative of 2 independent experiments. (C) Supernatant from parallel cultures were pooled and assessed in technical triplicates (*p<0.05) via ELISA for IL-13 and IFN-g.

In the current study we utilized bone marrow (BM) precursors to differentiate CD11b+ DC via standard GMCSF conditioning [20], [21], whereas CD103+ DC were differentiated via Flt-3 conditioned media (**Fig. 3a**), as previously described by Sathe et al [22]. DC were also magnetically sorted. Naïve hosts were adoptively transferred with T cells from OVA/alum-primed mice and subsequently engrafted with exogenously BM-derived and purified CD11b+ versus CD103+ DC prior to topical OVA challenge. To assess the in vivo functional role in triggering allergic T cell responses by respective engrafted DC subsets, harvested host LN were OVA stimulated in vitro for intracellular flow cytometry analyses of CD4+ expression of IL-4, IL-5, IL-13, and IFN-g (**Fig. 3b**). Using this approach, we found that CD11b+ DC led to increased allergic T cell responses, as indicated by elevated CD4+ IL-4+, IL-5+, IL-13+, and IFN-g+ frequencies (**Fig. 3b**). ELISA assays of select cytokines from supernatants of parallel run cultures also confirmed this (**Fig. 3c**).

**Figure 4:**
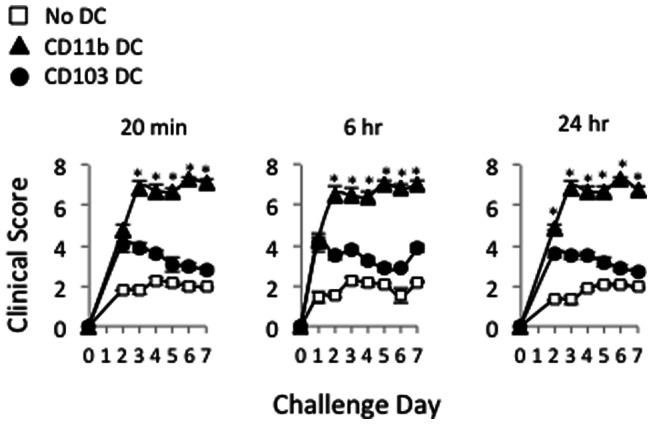
BM-derived CD11b+ DC engrafted into the conjunctiva leads to exacerbated ocular allergy clinical signs. Naïve hosts were adoptively transferred with T cells from OVA/alum-primed mice and hosts were subsequently engrafted with purified BM derived CD103+ or CD11b+ DC (1.0×10ˆ5 cells). OVA challenges were administered via ocular instillations once a day, and masked clinical scoring was performed once a day at 20 min, 6 hr and 24 hr post challenge. Statistical significance (*p<0.05) was derived from assessing CD11b+ vs. CD103+ DC clinical scores in an n = 4 per group. Data are representative of 2 independent experiments.

We also examined the role of CD11b+ vs. CD103+ DC in mediating clinical signs of ocular allergy, which include conjunctival chemosis, hyperemia, lid edema, and tearing/discharge [19]. To accomplish this, CD11b+ DC, CD103+ DC, or sham HBSS were engrafted into the conj of adoptively transferred hosts, as described above. Mice were challenged once daily for 10 d, and masked scoring was performed on each day at 20 min post challenge (i.e. consistent with the time frame for an immediate hypersensitivity response [19]), as well as 6 hr and 24 hr post challenge (i.e. consistent with the time frame for a late phase response [19]). Using this approach, we found increased clinical signs in response to engrafted CD11b+ DC, starting as early as challenge day 2 (**Fig. 4**). This was maintained through termination of the experiment on challenge day 7 (**Fig. 4**).

**Figure 5:**
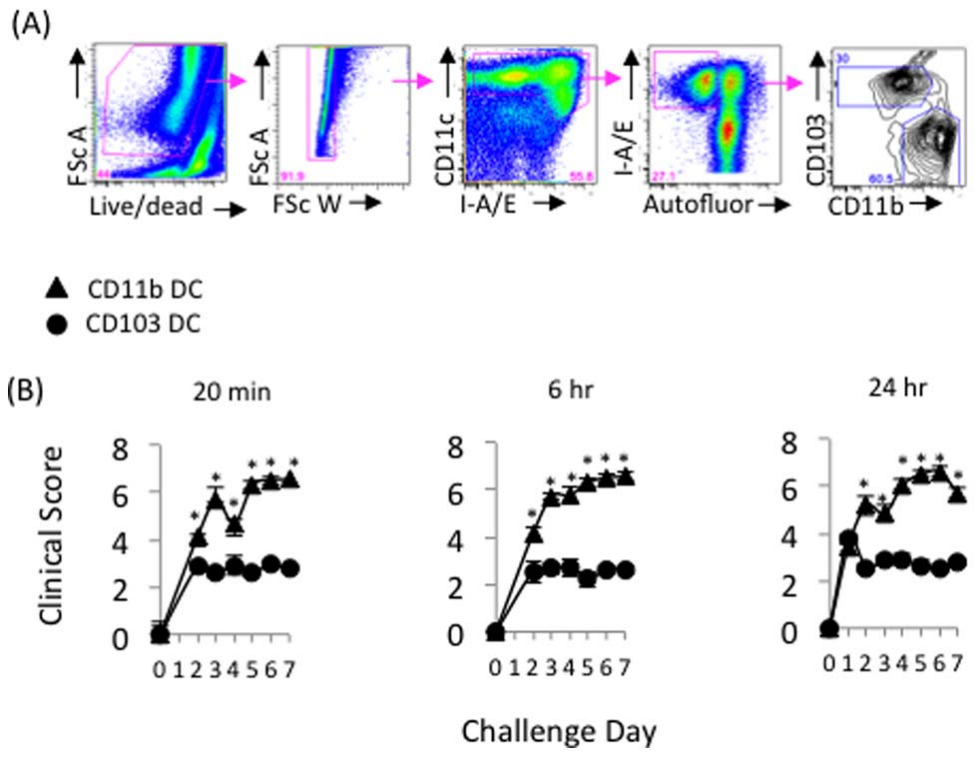
Lung CD11b+ DC engrafted heterotopically into the conjunctiva leads to exacerbated ocular allergy clinical signs. (**A**) Gating scheme for FACS sorting of lung CD11b+ vs. CD103+ DC. Single-cell suspensions of pooled lung (from n = 10 mice) gated for viability dye-, singlet, CD11c+, I-A/E+, and autofluorescent- cells, and CD11b+ vs. CD103+ were isolated. (**B**) Naïve host mice were adoptively transferred with T cells from OVA/alum primed mice and engrafted with purified lung CD103+ or CD11b+ DC (6.0×10ˆ4 cells). OVA challenges were administered via ocular instillations once a day, and masked clinical scoring was performed once a day at 20 min, 6 hr and 24 hr post challenge. Statistical significance (*p<0.05) was derived from assessing CD11b+ vs. CD103+ DC clinical scores in an n = 4 per group. Data are representative of 2 independent experiments.

We next determined whether these results were relevant with responses generated by already differentiated CD11b+ and CD103+ nonlymphoid tissue DC. To accomplish this, we FACS sorted lung CD11b+ vs. CD103+ DC (**Fig. 5a**) and heterotopically engrafted these respective subsets into the conj of adoptively transferred hosts. Clinical scores in response to instillation of OVA challenges were then ascertained. Interestingly, we found increased clinical signs in mice engrafted with purified lung CD11b+ DC, relative to mice engrafted with purified lung CD103+ DC (**Fig. 5b**). This was observed as early as challenge day 2 (**Fig. 5b**) and maintained through termination of the experiment on challenge day 7 (**Fig. 5b**).

## Discussion

Little is known about steady state DC subsets in the ocular mucosa, particularly regarding CD103+ and CD11b+ DC. One report by Ohbayashi et al identified via immunohistochemistry the presence of CD11b expressing DC in the normal mouse conjunctiva [17], [18]. Consistent with this, we found a population of CD11b+ DC, but we also identified another population, that are CD103+ DC. Interestingly, however, we found that CD103+ and CD11b+ DC play different roles in mediating adaptive immune responses. By using a previously described model of ocular allergy in which exogenous DC are engrafted into the ocular mucosa in topically challenged mice [19], we found that the CD11b+ DC subset was dominant in triggering allergic immune responses.

We were able to identify the presence of these DC in the ocular mucosa via direct comparison with CD103+ and CD11b+ DC previously described in the liver and lung [2]–[3]. Furthermore, in line with previous reports, we found that steady state CD11b+ DC in the ocular mucosa are F4/80+ and Sirp-a+, but negative to low for CD8a, DEC205, and langerin [2]–[4]. Conversely, we demonstrated that steady state CD103+ DC in the ocular mucosa are negative to low for F4/80 and Sirp-a, but CD8a+, DEC205+, and langerin+. In line with Edelson et al [4], we demonstrated that steady state CD11b+ DC in the ocular mucosa express significantly higher levels of Irf4, Csfr, and Cx3cr1. In contrast, steady state CD103+ DC in the ocular mucosa express significantly higher levels of Id2 and Flt3, as well as a marginal increase in Irf8. Finally, CD11b+ DC, but not CD103+, were readily detectible in the ocular mucosa of Flt3 KO mice.

To assess the functional role of these DC subsets in ocular allergy, we utilized our model previously described by Schlereth et al [19]. It involves adoptive transfer of T cells and topical OVA challenge in hosts that have been engrafted into the ocular mucosa with exogenously derived DC. Such site-specific engrafted DC were shown to capture topically instilled allergen and mobilize to eye-draining LN for Th cell stimulation [19]. This in turn leads to a robust augmentation of secondary allergic immune responses relevant in ocular allergy, which is absent when CCR7-/- DC are engrafted instead [19]. Thus, such an approach provides direct functional information of engrafted DC [19] and to this end was appropriate to query the role of CD11b+ vs. CD103+ DC in ocular allergy. Our approach revealed a dominant role for CD11b+ DC in triggering allergic immune responses in ocular allergy. This is supported by our identification in the eye-draining LN of increased Th cell responses, as well as increased clinical signs of ocular allergy (i.e. conjunctival hyperemia and chemosis, lid edema, and tearing/discharge [19]).

It is important to highlight, however, that our identification of a steady state CD11b+ DC dominance in the elicitation of allergic immune responses was only demonstrated here in a model of secondary immune responses. This is because CD11b+ or CD103+ DC were engrafted into mice adoptively transferred with OVA sensitized T cells, and consequent allergic responses to topical OVA challenges is predominantly due to reactivation of sensitized T cells [19]. Others have examined steady state CD103+ and/or CD11b+ DC in secondary allergic immune responses and corroborate our findings [13], [14]. Medoff et al adoptively transferred sensitized T cells into CD11b+ DC depleted mice (via use of CD11b-DTR mice), and found impaired allergic airway reactivity [13]. Likewise, Raymond et al reported a crucial role for CD11b+ DC in secondary allergic responses by testing allergic airway inflammation in sensitized mice [14]. In contrast, Nakano et al demonstrated a dominant role for pulmonary CD103+ DC in triggering Th2 responses to inhaled antigens [12]. However, their work was focused on primary immune responses. Thus, it may be surmised that CD103+ DC play a role in in allergic sensitization, whereas CD11b+ DC are key in secondary allergic reactions. Further work is needed to examine this.

Also requiring further investigation is the exact reasons by which CD11b+ DC are dominant in secondary allergic immune responses, as seen here and by others [13]–[14]. This is somewhat perplexing given the numerous reports converging on the inferiority of CD11b+ DC in mobilizing to draining LNs relative to their CD103+ counterparts [5], [6], [24]. In addition, endogenous CD11b+ DC normally reside deeper (i.e. in the stroma), and (as compared to intraepithelial CD103+ DC) would thus seem less advantageous with respect to allergen capture [23], [24]. On the other hand, as shown here and by others, endogenous CD11b+ DC are greater in absolute number than CD103+ DC [2]–[5], which could perhaps compensate for these deficits. Such differences in frequency, however, does not seem to play a particularly important role given that in the current study an equal number of CD103+ and CD11b+ DC were engrafted. Data from our study likewise put into question the importance of anatomic location given that both DC subsets were engrafted into the same space.

Also not offering a clear explanation as to the reasons for the dominant role for CD11b+ DC seen here, are other superior attributes previously described. CD11b+ DC are known to be proficient in phagocytosis, which could be advantageous with respect to allergen uptake [15]. CD11b+ DC are also poor at production of the Th1 polarizing cytokine, IL-12 [9]. However, such collective properties do not appear to be absolute as Nakano et al demonstrated that CD11b+ DC are inferior to their CD103+ counterparts in Th2 priming [12]. Thus future work is required to understand why CD11b+ DC are dominant in triggering secondary allergic immune responses in our model and others [13], [14].

In conclusion, we show here that steady state CD11b+ and CD103+ DC are present in the ocular mucosa; however, these DC have disparate roles in mediating adaptive immune responses. In a model of ocular allergy, wherein CD11b+ vs. CD103+ DC were engrafted into the ocular mucosa of mice adoptively transferred with sensitized T cells and challenged topically with allergen, revealed that CD11b+ DC are dominant in triggering secondary allergic immune responses relevant in ocular allergy. Future work is required to understand why CD11b+ DC are dominant in secondary allergic immune responses and whether such cells can be targeted therapeutically in treatment of atopic conditions such as allergic eye disease.

## Materials and Methods

### Mice and Anesthesia

Female C57BL/6 and Flt-3 knockout mice 8–12 wk old were purchased from Charles River Laboratories (Wilmington, MA) and Jackson laboratories (Bar harbor, ME). Mice were housed in a specific pathogen-free environment at the Schepens Eye Research Institute animal facility. The Institutional Animal Care and Use Committee approved all procedures. All animals were treated according to the ARVO Statement for the Use of Animals in Ophthalmic and Vision Research. Anesthesia was used for all surgical procedures with intraperitoneal administered ketamine/xylazine suspensions (120 and 20 mg/kg, respectively).

### Antibodies used for Flow Cytometry

For CD103 Ab we used clone 2E7 on PERCP-Cy5.5 (Biolegend). For CD11b Ab, we used clone M1/70 on PERCP-Cy5.5 (Biolegend), or PE (BD). For CD11c Ab, we used clone N418 on Alexa 488 or PE-Cy5 (Biolegend), or clone HL3 on PE-Cy7 (BD). For langerin (CD207) Ab we used clone eBioL31 on Alexa 647 (eBioscience). For CD45 Ab, we used clone 30-F11 on Alexa 488, PE, APC-Cy7 (BioLegend), or PE-Cy5 (BD). For DEC205 (CD205), we used clone NLDC-145 on PE-Cy7 (BioLegend). For F4/80 Ab, we used clone BM8 on APC-Cy7 (BioLegend). For I-A/I-E, we used clone M5/114.15.2 on PE-Cy7 or APC-Cy7 (BioLegend). For CD4, we used clone RM4-5 on PerCP-Cy5.5 (BioLegend); IL-4 on PE-Cy7 using clone 11B11 (BD Pharmingen); IL-5 on PE using clone TRFK5 (BioLegend); IL-13 on Alexa 647 using clone eBio13A (eBiosciences); and IFN-g on APC-Cy7 using clone XMG1.2 (BD Pharmingen). Cells were also stained with DAPI or fixable viability dye (eBioscience) for analysis of viable cells. All samples were pre-treated with purified Fc block (CD16/32) Ab clone 93 (eBioscience) according to manufacturer's instructions prior to staining. Aliquots were made for appropriate isotype controls.

### Enzymatic Digestion of Conjunctiva, Liver and Lung

This procedure has been previously described [16], [19]. Briefly, euthanized mice underwent transcardial perfusion with cold HBSS prior to collecting the organs assessed. For conjunctiva, all sites including bulbar through palpebral regions from both the superior and inferior areas were collected. Tissues were minced and digested in 2 mg/mL collagenase D (Roche, Indianapolis, IN) and 0.05 mg/mL DNase (Roche) for 2 to 3 hours at 37°C. Cell suspensions were then triturated in 20 mM EDTA and then passed through a 70-um filter (BD Falcon; Becton-Dickinson, Franklin Lakes, NJ). Cells were then thoroughly washed.

### Flow Cytometry Gating or Sorting for Phenotypic Analyses and qRTPCR

Viable cells from single cell suspensions were enumerated via trypan blue exclusion assay, and subsequently stained for CD11c, I-A/E, CD11b, CD103 and live/dead, then washed thoroughly. CD11b+ DC were viability dye- CD11c+ I-A/E+ CD11b hi autofluorescence- (i.e., empty FITC channel), CD103-. In contrast, CD103+ DC were viability dye- CD11c+ I-A/E+ CD11b-/low autofluorescence- CD103+.

### qRTPCR

Purified cells obtained via FACS sorting were used. The differential expression of selected genes was verified by using quantitative real-time PCR (qPCR) procedures. Total RNA was isolated using Trizol (Invitrogen) and RNeasy Microkit (Qiagen). The first strand complementary DNAs (cDNA) were transcribed by employing SuperScript III Reverse Transcriptase (Invitrogen, Grand Island, NY) and random hexamer primers (Invitrogen). Quantitative real-time polymerase chain reaction was performed in duplicates of the same concentration of cDNAs using Taqman Universal PCR Mastermix and FAM-MGB dye-labeled predesigned primers and probes (Applied Biosystems, Inc., Foster City, CA) for Interferon regulatory factor 8 (Irf8) (Mm01250091_m1), Interferon regulatory factor 4 (Irf4) (Mm00516434_m1), Inhibitor of DNA binding 2 (ID2) (Mm00711781_m1), FMS-like tyrosine kinase 3 (Flt3) (Mm00439000_m1), Colony stimulating factor 3 receptor (granulocyte) (Csfr) (Mm01266656_g1), Chemokine (C-X3-C) receptor 1 (Cx3cr1) (Mm02620111_s1) and as the endogenous control, the Glyceraldehyde 3-phosphate dehydrogenase (GAPDH) (Mm99999915_g1). Differential gene expression was calculated according to the

Comparative Ct method outlined in Applied Biosystems User Bulletin 2 (updated 2001). The relative expression level of each sample was expressed as fold change from naïve control.

### Generation of Bone Marrow (BM)-Derived DC Subsets

BM derived DC were generated as previously described [19]–[21]. Briefly, femurs and tibiae were collected from freshly euthanized C57BL/6 mice. BM cells were seeded at 2×10ˆ5/ml in RPMI 1640 (BioWitthaker, Walkersville, MD) supplemented with 10% FBS, 1% penicillin/streptomycin, plus 20 ng/ml mouse GM-CSF (Biolegend, San Diego, USA) at 37°C with 5% carbon dioxide for 7 days. Additional GM-CSF was added on day 4 (20 ng/ml). Generation of CD103+ DC was previously described [22]. Briefly, Flt-3 (100 ng/ml, eBioscience) was used in place of GM-CSF and cultured for 7 days [22]. To increase the number of CD103+ DC, additional GM-CSF (20 ng/ml) was added to plates on day 4 [22]. Further enrichment of CD11b+ and CD103+ subsets was accomplished via magnetic sorting, as detailed in section below.

### MACS Sorting of BM-Derived DC Subsets used for Engraftment

For BM-derived DC, cells were harvested on day 7 of cultures and prepared for magnetic sorting according to manufacturer's instructions (Miltenyi Biotec, Auburn, USA). For CD11b+ DC, GM-CSF cultivated cells were MACS sorted using anti-CD11c beads alone, as all CD11c+ cells co-express CD11b+. For CD103+ DC, Flt-3 cultivated cells were sorted using anti-CD103 beads, as all CD103+ cells co-express CD11c+; <90% purity was achieved for both subsets. Purified cells were washed thoroughly and prepared for subconjunctival injection.

### FACS Sorting of Lung DC Subsets used for Engraftment

Following enzymatic digestion, DC were first purified magnetically via anti-CD11c beads, according to manufacture's instructions (Miltenyi Biotec). Cells were then thoroughly washed and enumerated. Cells were then stained for FACS sorting as detailed above.

### T Cell Adoptive Transfer

This method in allergic eye disease has been previously described [19]. Briefly, T cells were obtained from donor wild-type (WT) C57BL/6 mice that were immunized once intraperitoneal with a 100 ul suspension containing 1 mg aluminum hydroxide (Sigma Aldrich, St. Louis, USA) diluted in HBSS, 300 ng pertussis toxin (Sigma Aldrich), and 100 ug ovalbumin (Sigma Aldrich). Donor mice 2 wk post immunization were euthanized and spleens were collected. Donor spleens were prepared into single-cell suspensions by tissue press using a sterile 70 um sieve, and cells were then treated with red blood cell lysis buffer according to manufacturer's instructions (Sigma Aldrich) and washed thoroughly. Donor T cells were enriched via MACS sorting using anti-CD90.2 Ab according to manufacturer's instructions (Miltenyi Biotec, Auburn, USA). The sorted donor population was then enumerated via trypan blue exclusion assay, and donor T cells were set at a concentration 1×10ˆ7/ml of sterile HBSS. Recipient mice were adoptively transferred IV with 4×10ˆ6 donor T cells.

### Conjunctival Engraftment and Ocular Allergy

This model was previously described [19]. Briefly OVA primed T cells were prepared as described above, and 4×10ˆ6 T cells were adoptively transferred into naïve hosts. Host mice were then anesthetized 16 hr later for unilateral injection of cells into the ocular mucosa by way of subconjunctival injection. Injection volume was 10 ul of sterile HBSS and contained 1×10ˆ5 purified BM derived CD11b+ or CD103+ DC. In another experiment, mice were similarly injected with 6×10ˆ4 purified CD11b+ or CD103+ DC from enzymatically digested lungs from donor mice. Challenge via topical OVA instillation (250 ug/5 ul eye drop) was administered immediately following subconjunctival (SCJ) injection, and then challenged additionally (to account for significant tearing post SCJ injection) twice more in 20 minute intervals. Challenges were subsequently administered once daily, for 7 days.

### Clinical Scoring

This procedure has been described previously [19] and performed here in a masked fashion by two independent observers. Briefly, scoring was performed 20 min post challenge and done once daily from day 1 (i.e. 24 hr following SCJ injection) to day 7. Mice where examined biomicroscopically based on four independent parameters, which include: 1) lid swelling; 2) tearing; 3) chemosis; and 4) conjunctival vasodilation (redness). Each parameter was ascribed 0 (i.e. absent) to 3+ points (i.e. maximal) and were summed to yield a maximum score of 12+.

### T cell Cytokine Analyses

Regional LN (cervical and submandibular) were collected and pooled from freshly euthanized mice. Single-cell suspensions were prepared and enumerated via trypan blue exclusion assay. Cells were plated in round-bottom 96-wells at a concentration of 2.0×10ˆ6/ml in triplicate wells of RPMI (10% FBS) with OVA (1 mg/ml) for up to 72 hr and restimulated with PMA/Ionomycin with Golgiplug (BD Pharmingen) for up to 4 hours. Cells were harvested and stained for extracellular CD4, and intracellular IL-4, IL-5, IL-13, and IFN-g. Parallel cultures were established for ELISA analyses of culture supernatant, and thus restimulated with PMA/Ionomycin only. ELISAs (eBioscience) for IFN-g and IL-13 were analyzed.

### Statistical analysis

Statistical analyses included 1-way ANOVA and Bonferroni's Multiple Comparison Test, in addition to two-tailed student's t-test. Standard error and standard deviation of the mean were calculated. A *p*-value <0.05 was considered statistically significant.

## References

[1] Hashimoto D, Miller J, Merad M (2011) Dendritic cell and macrophage heterogeneity in vivo. Immunity 23 35(3): 323–35. 10.1016/j.immuni.2011.09.007 PMC4520532 21943488

[2] Ginhoux F, Liu K, Helft J, Bogunovic M, Greter M, et al (2009) The origin and development of nonlymphoid tissue CD103+ DC. J Exp Med 21 206(13): 3115–30. 10.1084/jem.20091756 PMC2806447 20008528

[3] Bogunovic M, Ginhoux F, Helft J, Shang L, Hashimoto D, et al (2009) Origin of the lamina propria dendritic cell network. Immunity 18 31(3): 513–25. 10.1016/j.immuni.2009.08.010 PMC2778256 19733489

[4] Edelson BT, KC W, Juang R, Kohyama M, Benoit LA, et al (2010) Peripheral CD103+ dendritic cells form a unified subset developmentally related to CD8alpha+ conventional dendritic cells. J Exp Med 12 207(4): 823–36. 10.1084/jem.20091627 PMC2856032 20351058

[5] Varol C, Vallon-Eberhard A, Elinav E, Aychek T, Shapira Y, et al (2009) Intestinal lamina propria dendritic cell subsets have different origin and functions. Immunity 18 31(3): 502–12. 10.1016/j.immuni.2009.06.025 19733097

[6] Ho AW, Prabhu N, Betts RJ, Ge MQ, Dai X, et al (2011) Lung CD103+ dendritic cells efficiently transport influenza virus to the lymph node and load viral antigen onto MHC class I for presentation to CD8 T cells. J Immunol 1 187(11): 6011–21. 10.4049/jimmunol.1100987 22043017

[7] D′Agostino PM, Kwak C, Vecchiarelli HA, Toth JG, Miller JM, et al (2012) Viral-induced encephalitis initiates distinct and functional CD103+ CD11b+ brain dendritic cell populations within the olfactory bulb. Proc Natl Acad Sci U S A 17 109(16): 6175–80. 10.1073/pnas.1203941109 PMC3340999 22474352

[8] Bedoui S, Whitney PG, Waithman J, Eidsmo L, Wakim L, et al (2009) Cross-presentation of viral and self antigens by skin-derived CD103+ dendritic cells. Nat Immunol 10(5): 488–95. 19349986 10.1038/ni.1724

[9] Maldonado-López R, Maliszewski C, Urbain J, Moser M (2001) Cytokines regulate the capacity of CD8alpha(+) and CD8alpha(-) dendritic cells to prime Th1/Th2 cells in vivo. J Immunol 15 167(8): 4345–50. 10.4049/jimmunol.167.8.4345 11591758

[10] Desch AN, Randolph GJ, Murphy K, Gautier EL, Kedl RM, et al (2011) CD103+ pulmonary dendritic cells preferentially acquire and present apoptotic cell-associated antigen. J Exp Med 29 208(9): 1789–97. 10.1084/jem.20110538 PMC3171085 21859845

[11] McDole JR, Wheeler LW, McDonald KG, Wang B, Konjufca V, et al (2012) Goblet cells deliver luminal antigen to CD103+ dendritic cells in the small intestine. Nature 14 483(7389): 345–9. 10.1038/nature10863 PMC3313460 22422267

[12] Nakano H, Free ME, Whitehead GS, Maruoka S, Wilson RH, et al (2012) Pulmonary CD103(+) dendritic cells prime Th2 responses to inhaled allergens. Mucosal Immunol 5(1): 53–65. 22012243 10.1038/mi.2011.47 PMC3697034

[13] Medoff BD, Seung E, Hong S, Thomas SY, Sandall BP, et al (2009) CD11b+ myeloid cells are the key mediators of Th2 cell homing into the airway in allergic inflammation. J Immunol 1 182(1): 623–35. 10.4049/jimmunol.182.1.623 PMC2718444 19109196

[14] Raymond M, Rubio M, Fortin G, Shalaby KH, Hammad H, et al (2009) Selective control of SIRP-alpha-positive airway dendritic cell trafficking through CD47 is critical for the development of T(H)2-mediated allergic inflammation. J Allergy Clin Immunol 124(6): 1333–42. 19748659 10.1016/j.jaci.2009.07.021

[15] Dudziak D, Kamphorst AO, Heidkamp GF, Buchholz VR, Trumpfheller C, et al (2007) Differential antigen processing by dendritic cell subsets in vivo. Science 5 315(5808): 107–11. 10.1126/science.1136080 17204652

[16] Hattori T, Chauhan SK, Lee H, Ueno H, Kaplan DH, et al (2011) Characterization of Langerin-expressing dendritic cell subsets in the normal cornea. Invest Ophthalmol Vis Sci 28 52(7): 4598–604. 10.1167/iovs.10-6741 PMC3175952 21482644

[17] Ohbayashi M, Manzouri B, Flynn T, Toda M, Ikeda Y, et al (2007) Dynamic changes in conjunctival dendritic cell numbers, anatomical position and phenotype during experimental allergic conjunctivitis. Exp Mol Pathol 83(2): 216–23. 17560570 10.1016/j.yexmp.2007.04.007

[18] Manzouri B, Flynn T, Ohbayashi M, Ono SJ (2008) The dendritic cell in allergic conjunctivitis. Ocul Surf 6(2): 70–8. 18418504 10.1016/s1542-0124(12)70270-7

[19] Schlereth S, Lee HS, Khandelwal P, Saban DR (2012) Blocking CCR7 at the Ocular Surface Impairs the Pathogenic Contribution of Dendritic Cells in Allergic Conjunctivitis. Am J Pathol 180(6): 2351–60. 22507838 10.1016/j.ajpath.2012.02.015 PMC5691338

[20] Lutz MB, Kukutsch N, Ogilvie AL, Rössner S, Koch F, et al (1999) An advanced culture method for generating large quantities of highly pure dendritic cells from mouse bone marrow. J Immunol Methods 1 223(1): 77–92. 10.1016/s0022-1759(98)00204-x 10037236

[21] Hattori T, Saban DR, Emami-Naeini P, Chauhan SK, Funaki T, et al (2012) Donor-derived tolerogenic dendritic cells suppress immune rejection in the indirect allosensitization-dominant setting of corneal transplantation. J Leukoc Biol 91(4): 621–7. 22291211 10.1189/jlb.1011500 PMC3317274

[22] Sathe P, Pooley J, Vremec D, Mintern J, Jin JO, et al (2011) The acquisition of antigen cross-presentation function by newly formed dendritic cells. J Immunol 1 186(9): 5184–92. 10.4049/jimmunol.1002683 21422244

[23] Bogunovic M, Ginhoux F, Helft J, Shang L, Hashimoto D, et al (2009) Origin of the lamina propria dendritic cell network. Immunity 18 31(3): 513–25. 10.1016/j.immuni.2009.08.010 PMC2778256 19733489

[24] Schulz O, Jaensson E, Persson EK, Liu X, Worbs T, et al (2009) Intestinal CD103+, but not CX3CR1+, antigen-sampling cells migrate in lymph and serve classical dendritic cell functions. J Exp Med 21 206(13): 3101–14. 10.1084/jem.20091925 PMC2806467 20008524

